# Did social media interaction replace quantitatively and qualitatively social face to face interaction during first months of COVID-19 pandemic ?

**DOI:** 10.1192/j.eurpsy.2021.714

**Published:** 2021-08-13

**Authors:** A. Grech

**Affiliations:** Mount Carmel Hospital, Mental Health Services, Attard, Malta

**Keywords:** social media, COVID- 19, psychological wellbeing, online interaction

## Abstract

**Introduction:**

COVID-19 has changed entire lives. Lockdowns all over the world have resulted in social isolation and whole populations started to live a new social norm.

**Objectives:**

In this presentation I will discuss the role social media has played in social interactions during lockdown. Mainly if social media use has replaced social face to face interactions. This both in terms of quantity and emotional quality of interactions. I will be using a study we conducted in Malta in this regard.

**Methods:**

It seems that social media use has increased during lockdown. To analyse this in Malta we studied a dataset comprising more than 50,000 comments and more than 150,000 interactions that has been extracted from Facebook covering a time span of four months. These months were the first wave of COVID- 19 cases in Malta. We conducted a quantitative and qualitative data analysis to establish the social polarity of the content.

**Results:**

I will discuss how social media has in part replaced the social face to face interactions during COVID- 19 pandemic initial months, and use the results of the study we conducted in Malta as an example.

**Conclusions:**

It would be useful to analyse further the use of social media in the new social norm caused by COVID- 19. Most importantly how it could be used to decrease social isolation effectively and increase psychological wellbeing.

